# The Evaluation of Changing the Eponym Churg–Strauss Syndrome Due to the 2012 Revised International Chapel Hill Consensus Conference Nomenclature of Vasculitides

**DOI:** 10.3390/jcm13123424

**Published:** 2024-06-11

**Authors:** Gokhan Sargin

**Affiliations:** Department of Rheumatology, Medical Faculty, Aydin Adnan Menderes University, 09010 Aydin, Turkey; gokhan_sargin@hotmail.com

**Keywords:** Churg–Strauss syndrome, eosinophilic granulomatosis with polyangiitis, vasculitides, eponym, rheumatic diseases

## Abstract

**Background:** Eponyms do not describe any pathogenesis of a disease. So, there is no other way than to memorize the disease or anatomical area. Over the years, new nomenclatures have been suggested for some diseases due to a better understanding of the pathogenesis. In this article, the changes in the use of Churg–Strauss syndrome were investigated. **Methods:** In the study, a computerized search was performed using the PubMed database. Books and documents, clinical trials, editorials, meta-analyses, reviews, and case reports were included in the study. Data were obtained from the title of the database, and the variations or distribution by year for the nomenclature of the most related studies were evaluated. **Results:** Overall, 68.3% of the articles included CSS, 25.7% included eosinophilic granulomatous polyangiitis (EGPA), and 6.0% included both nomenclatures. When evaluated in terms of the distribution according to years, it was determined that there was a statistically significant increase in use in terms of EGPA. When evaluated among specific section journals, the highest rate was in Rheumatology (29.4%). The highest rate of using CSS was in the Rheumatology (25.1%) journals, followed by Pulmonary/Respiratory (17%), Cardiovascular (12%), and Allergy/Immunology/Biology (9.8%). The use of EGPA combined with CSS decreased in all the specific journals from 2012 to the present. **Conclusions:** The findings of the study revealed that the number of articles with the eponym of EGPA showed an increased frequency in contrast to a decreasing frequency for those with CSS during recent years. Today, with the elaboration of the disease pathogenesis and the increase in knowledge, the trend has shifted in this direction.

## 1. Introduction

In the field of medicine, people who describe or find any disease, syndrome, or anatomical structure have made their names [1,2]. Eponyms are a tradition used in medicine and honor the scientist who played an important role in defining the disease [2]. These nomenclatures used do not describe any pathogenesis of the disease. In this case, it seems that there is no other way than to memorize the disease or anatomical area. Eponyms developed from the end of the 19th century to the beginning of the 20th century. Moreover, they often reflected the dominance of the scientific cultures and languages of that period [2]. These names are frequently encountered in daily life. For example, Sjogren’s syndrome, Crohn’s disease, and Alzheimer’s disease are some of those diseases that can be counted. There is no doubt that these scientists have made great contributions to medical literature and its development.

Antineutrophil cytoplasmic antibody (ANCA)-associated vasculitides, which are small-scale vessel vasculitides, consist of granulomatous polyangiitis, microscopic polyangiitis, and eosinophilic granulomatous polyangiitis (Churg–Strauss). It is a granulomatous vasculitis with prominent lung involvement and affects many organ systems. Although its etiology is not known exactly, it is associated with allergy and atopic disorders, and most of the cases have a history of allergic rhinitis, asthma, and eosinophilia. Radiologically migratory infiltrates, non-cavitational nodular infiltrates, and diffuse interstitial lung disease are the main pulmonary manifestations of this vasculitis. The difference from granulomatosis polyangiitis is that no destructive respiratory tract damage is observed. In addition, neurological symptoms, cardiac symptoms, and skin findings are also rarely observed [3,4,5].

Except for eosinophilia, approximately one-third of the patients with this vasculitis are positive for ANCAs (5). Necrotizing vasculitis, eosinophilic tissue infiltration, and extravascular granuloma formation constitute the pathological findings. The vasculitis is diagnosed based on asthma, eosinophilia, neuropathy, pulmonary infiltrates, paranasal sinus abnormality, and eosinophilic tissue infiltration findings in the biopsy [4,5]. The disease is managed with a multisystem approach including rheumatology, dermatology, pulmonology, neurology, and nephrology. Although corticosteroids alone are sufficient in the treatment, immunosuppressive agents can be applied in gastrointestinal, cardiac, and central nervous system involvement [3,4,5].

At the first International Chapel Hill Consensus Conference on the Nomenclature of Systemic Vasculitides held in 1994, a consensus was reached on the names of common forms of vasculitis, and definitions were created for each [6]. This classification, which is currently widely accepted, was revised in 2012 due to advances in the pathogenesis of vasculitis and to create additional categories [3]. At the 2012 International Chapel Hill Consensus Conference, the use of eponyms in the naming of vasculitides began to be discontinued, but eponyms were retained in cases where an alternative name could not be found and the pathophysiology was insufficient. One of these nomenclatures is Churg–Strauss syndrome, which was small-vessel vasculitis at the Chapel Hill Consensus Conference in 1994. The syndrome was reported as eosinophilic granulomatosis with polyangiitis (Churg–Strauss) at the 2012 International Chapel Hill Consensus Conference [3].

Over time, different uses have occurred in the literature, including both nomenclatures. Tabb ES, et al. reviewed the diagnostic criteria for the change from CSS to EGPA in the literature [7]. However, there is no study in the literature that reflects the data on the change. This study aims to analyze the frequency of the differences in the use of Churg–Strauss syndrome, eosinophilic granulomatosis with polyangiitis (Churg–Strauss), or both of these from the past to the present.

## 2. Materials and Methods

In the study, a search was performed using the PubMed database from the study titled Allergic Granulomatosis, Allergic Angiitis, and Periarteritis Nodosa published in the American Journal of Pathology in 1951 by Jacob Churg and Lotte Strauss to date [8]. The following keywords were searched from the literature: “Churg-Strauss syndrome”, Churg-Strauss” (CSS), “Eosinophilic granulomatosis with polyangiitis”, and “Eosinophilic granulomatosis polyangiitis”.

Books and documents, clinical trials, editorials, meta-analyses, reviews, and case reports in the PubMed database were included in the study. Data were obtained from the title of the database, and the variations or distribution by year for the nomenclature of the most related studies were evaluated. Ethics committee approval was not obtained because the data included any title obtained from the database with open access.

The analysis of the data was carried out using SPSS for Windows version 21.0 (Statistical Package for Social Sciences Inc., Chicago, IL, USA). The data were presented as frequency (n) and percentage (%). Pearson’s chi-squared test, Yates’ chi-squared test, and Fisher’s exact test were used for proportions to analyze the difference in the number of publications that used the eponym without the representative name. The *p*-value of <0.05 was considered statistically significant.

## 3. Results

**Figure 1:**
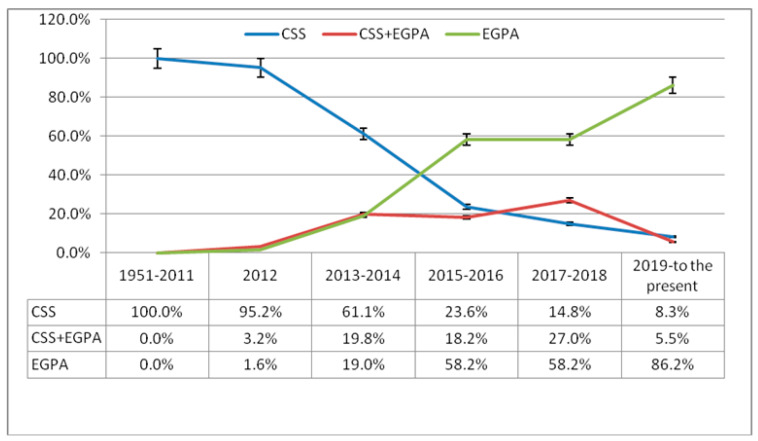
Distribution of the number of publications of “Churg-Strauss Syndrome” and “Eosinophilic Granulomatous Polyangiitis”, and both of these in the title according to years.

A total of 1595 publications were reviewed in the PubMed database from 1951 to the present, and 1089 (68.3) of the articles included CSS, 410 (25.7%) of the articles included EGPA, and 96 (6.0%) of all the publications included both. While 99.7% of all the articles included in the study between 1951 and 2012 included the nomenclature as CSS, this rate decreased to 22.4% since 2013. Since 2013, EGPA has been used in 63.1% of all the articles, while CSS and EGPA have been used together in 14.5%. The nomenclature for CSS was used in sixty (95.2%) of all the articles included in the study in 2012, EGPA in one (1.6%), and CSS and EGPA in two (3.2%) articles. When evaluated in terms of the distribution according to years, it was determined that there was a statistically significant increase in use in terms of EGPA, while there was a significant decrease in CSS. Also, the rate of combination use of CSS and EGPA decreased (Table 1) (Figure 1).

**Figure 2:**
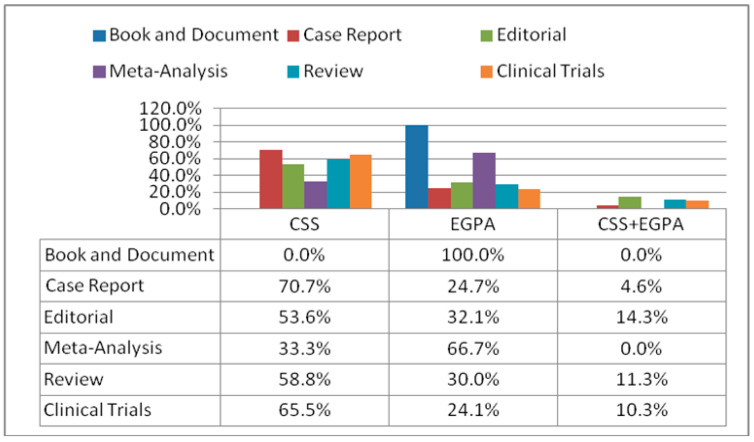
The distribution of the eponyms “Churg-Strauss” and “Eosinophilic granulomatosis with polyangiitis”, and both of these by the article types.

When evaluating all the articles, there were twelve-hundred-seventy-seven (80.1) case reports, two-hundred-fifty-seven (16.1%) reviews/case-based reviews, twenty-nine (1.8%) clinical trials, twenty-eight (1.8%) editorials, three (0.2%) meta-analyses, and only one (0.1%) book. Among these groups, the case reports and reviews/case-based reviews were significantly higher than the others. The distribution of the eponyms CSS, EGPA, and both of these by the article type is shown in Figure 2.

**Figure 3:**
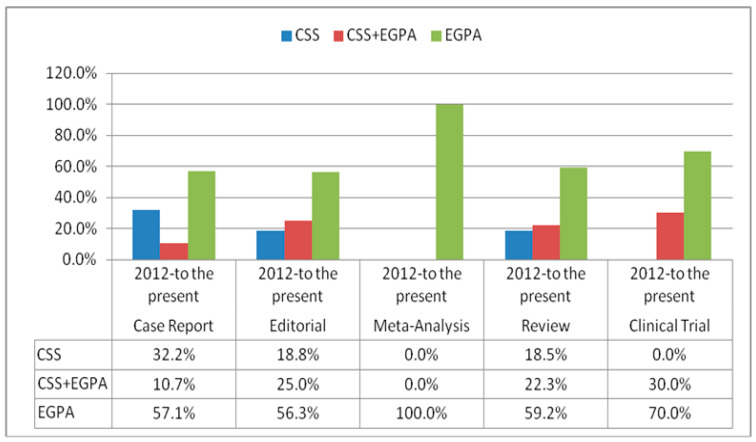
The distribution of eponyms “Churg-Strauss Syndrome” and “Eosinophilic granulomatosis with polyangiitis”, and both of these according to the article types from 2012 to the present.

A total of 410 (57.7%) articles were found in searches that included EGPA in their title since 2012. Until 2012, seven-hundred-twenty-five (82%) case reports, one-hundred-twenty-seven (14.4%) reviews, nineteen (2.1%) clinical trials, and twelve (1.4%) editorials were published with the eponym of CSS. When evaluated from 2012 to the present, it was determined that the amount of case reports, editorials, meta-analyses, reviews, and clinical studies in which EGPA was used in the article title was numerically higher than CSS and CSS+EGPA. The distribution of the eponyms according to the article style from 2012 to the present is shown in Figure 3.

When evaluated among specific section journals, the highest rate was in Rheumatology (29.4%). The ratios of the other journals were Pulmonary/Respiratory (15.7%), Allergy/Immunology/Biology (13.1%), Cardiovascular (11.3%), Dermatology (7.9%), Neurology (8.3%), Ophthalmology (4.2%), Gastroenterology (3.8%), Nephrology (3.2%), and Pathology (3.1%), respectively. Those most frequently titled CSS were in the Rheumatology (25,1%) journals, followed by Pulmonary/Respiratory (17%), Cardiovascular (12%), and Allergy/Immunology/Biology (9.8%). The use of EGPA in the titles has increased proportionally compared to the other nomenclatures since 2012. The differences in the nomenclature according to the years and different specific journals are shown in Table 2.

## 4. Discussion

The use of the eponym CSS alone decreased from the 2012 revised International Chapel Hill Consensus Conference nomenclature of vasculitides to the present. It was also observed that there was a greater tendency to use EGPA in titles than to use only CSS. There is no established rule for developing eponyms, and it is estimated that there are more than 8000 eponyms today [2,9]. Some of these eponyms can be single-name and some can be multi-name, like Churg–Strauss. Disease names and definitions have changed over time as medical knowledge has developed and progressed. Based on this, some nomenclature in the International Chapel Hill Consensus Conference 2012 has changed, unlike the International Chapel Hill Consensus Conference 1994 [3,6]. The aim here was to make naming more understandable and valuable based on new developments and understandings in disease symptoms, mechanisms, and pathogenesis. Eosinophilic granulomatosis with polyangiitis (Churg–Strauss) was defined in the International Chapel Hill Consensus Conference 2012 as eosinophil-rich inflammation often affecting the airways and predominantly small to medium vessels and associated with asthma and eosinophilia and necrotizing granulomatous inflammation [3]. The prominence of eosinophils in the blood and tissue is an essential feature of this vasculitis and thus is highlighted in the name. The eponym CSS was replaced by EGPA in part to achieve nomenclature symmetry with MPA and GPA. In a study evaluating the change from Wegener to granulomatosis with polyangiitis, a significant difference was found in the number of publications using only the Wegener eponym [10]. This study determined that, since the naming change, the use of the CSS name alone in titles has decreased proportionally compared to others.

Jacob Churg was a valuable and pioneering scientist involved with studies on kidney diseases, the interpretation of kidney biopsies, and lung, pleural, and vascular diseases. He developed new techniques for the understanding, study, and interpretation of many kidney diseases. These studies include diseases such as lupus nephritis, diabetes mellitus, hemolytic uremic syndrome, and amyloidosis. He has made enormous contributions to the understanding of kidney pathology and nephrology. Apart from the above, he is known especially for his work on allergic granulomatosis (Churg–Strauss syndrome) with Lotte Strauss. Dr. Lotte Strauss is a pioneer of perinatal pathology and is accepted as one of the most important founders of the pathology. She has studied the placental structure, Farber’s disease, and childhood leukemia [11,12,13].

Churg and Strauss described cases of fever, asthma, hypereosinophilia, and histopathologically nodular swellings around the small arteries of many organs [8]. They reported in their later publication that the lung is not the best place to look for allergic granulomas, noting that allergic granulomas tend to occur more frequently in the heart, liver, spleen, skin, and walls of blood vessels [14]. However, they used the term “allergic granulomatosis and angiitis” to distinguish the disease from PAN in the first cases they described [8]. In this article, it has been suggested that other allergic syndromes may represent benign forms of allergic granulomatosis, while angiitis is the most malignant expression. Lanham et al. used the terminology Churg–Strauss syndrome and reported the criteria to include asthma, peripheral eosinophilia, rash, neuropathy, hypertension, arthritis, or myalgia [15]. The diagnosis of EGPA mainly occurs clinically. This disease has low mortality compared to other ANCA-associated vasculitides. However, organ damage, relapses, and exacerbations can disrupt patients’ quality of life [16]. Glucocorticoids, azathioprine, cyclophosphamide, rituximab, and mepolizumab are among the treatment regimens [17]. The American College of Rheumatology developed classification criteria to include biopsies showing asthma, eosinophilia, neuropathy, unstable pulmonary infiltrate, paranasal sinus abnormality, and extravascular eosinophils [18]. Subsequently, at the revised International Chapel Hill Consensus Conference in 2012, small-vessel vasculitides were defined as ANCA-associated and immune complex vasculitides [3]. Moreover, it has been defined as a syndrome by designating eosinophilic granulomatous polyangiitis (Churg–Strauss). The sensitivity was 85% (95% CI 77% to 91%) and the specificity was 99% (95% CI 98% to 100%) for the classification set for the vasculitis by the 2022 American College of Rheumatology/European Alliance of Associations for Rheumatology Classification Criteria [19]. Today, the use of the disease name EGPA has been widely accepted. The limitation of the study is that only one database was searched and only books and documents, case reports, clinical trials, editorials, meta-analyses, and reviews were evaluated. Additionally, recent articles may have been added to the search database after the date we analyzed the study data.

## 5. Conclusions

The descriptive term appears to be gaining traction and replacing the eponym. The findings of this study revealed that the number of articles with the eponym of EGPA showed an increasing frequency in contrast to a decreasing frequency for those with CSS during recent years. Today, with the elaboration of the disease pathogenesis and the increase in knowledge, the trend has shifted in this direction. This situation has become more evident, especially with a better understanding of ANCAs’ place in the pathogenesis. It is very important in terms of development that every scientist who has contributed to the medical literature is honored and valued under all conditions.

## Figures and Tables

**Table 1:** Distribution of the number of publications of “Churg-Strauss Syndrome” and “Eosinophilic Granulomatous Polyangiitis”, and both of these in the title according to years.

	CSS	EGPA	CSS+EGPA	Total
**1981–1990**	95 (100%)	-	-	95 (100%)
**1991–2000**	260 (100%)	-	-	260 (100%)
**2001–2011**	519 (100%)	-	-	519 (100%)
**2012**	60 (95.2%)	1 (1.6%)	2 (3.2%)	63 (100%)
**2013–2016**	103 (43.6%)	88 (37.3%)	45 (19.1%)	236 (100%)
**2017–2020**	26 (10.5%)	178 (72.1%)	43 (17.4%)	247 (100%)
**2021 to the present**	16 (9.7%)	143 (86.7%)	6 (3.6%)	165 (100%)

**Table 2:** The different nomenclature according to the years and different specific journals.

	1951–2011	2012 to the Present		
	CSS	CSS	CSS+EGPA	EGPA
**Allergy/Immunology/Biology**	61 (10.6%)	8 (6.3%)	16 (24.6%)	50 (18.7%)
**Dermatology**	43 (7.5%)	13 (10.3%)	10 (15.4%)	16 (6.0%)
**Pulmonary/Respiratory**	109 (19%)	10 (7.9%)	7 (10.8)%	36 (13.5%)
**Rheumatology**	162 (28.2%)	14 (11.1%)	20 (30.8%)	108 (40.4%)
**Pathology**	27 (4.7%)	2 (1.6%)	-	3 (1.1%)
**Cardiovascular**	51 (8.9%)	33 (26.2%)	4 (6.2%)	29 (10.9%)
**Neurology**	50 (8.7%)	19 (15.1%)	5 (7.7%)	12 (4.5%)
**Nephrology**	19 (3.3%)	8 (6.3%)	-	6 (2.2%)
**Gastroenterology**	27 (4.7%)	4 (3.2%)	2 (3.1%)	6 (2.2%)
**Ophthalmology**	26 (4.5%)	15 (11.9%)	1 (1.5%)	1 (0.4%)

## Data Availability

Data are available from the corresponding author upon reasonable request.
